# Evaluation of the Extended Induction Programme for International Medical Graduate Core Psychiatry Trainees in CNWL

**DOI:** 10.1192/bjo.2024.315

**Published:** 2024-08-01

**Authors:** Sara Moghrabi, Saikat Roy, Bartlomiej Matras

**Affiliations:** CNWL, London, United Kingdom

## Abstract

**Aims:**

International Medical Graduate (IMG) doctors make up a significant proportion of the British medical workforce. In 2022, 26% of doctors in training graduated outside the UK. Psychiatry was one of the most frequently chosen specialties by IMGs. Doctors joining the NHS face several challenges such as differential attainment and discrimination. Increasing recognition of such issues led to the recent publication of a national guidance for IMG induction. In 2021, CNWL appointed an IMG lead to design and implement a comprehensive induction and ongoing support programme for all IMG doctors joining the trust. The first induction for new IMG core trainees consisted of six sessions and included a mixture of communication skills workshops and tutorials. It started in February 2021 and was delivered over four weeks. Since then, the programme has run twice a year with each intake of new core trainee doctors.

**Methods:**

Nineteen CNWL IMG core trainees who participated in the induction programme between 2021 and 2023 were invited to complete an online survey. The data was collected between December 2023 and January 2024. It consisted of Likert scale ratings of the content of the programme, its relevance, and its impact on trainees’ confidence. The usefulness of each session was also measured. Trainees were encouraged to provide free-text comments with suggestions for improvements.

**Results:**

Sixteen out of nineteen trainees submitted responses. There was a consensus amongst all trainees that the induction covered essential topics. Fifteen out of sixteen participants felt more confident in their role after the sessions. The first communication skills workshop covering history taking and mental state examination was considered to be the most useful with twelve participants rating it as excellent. The workshop on managing conflict with simulation scenarios was ranked second most helpful. Tutorials on NHS structure and a training portfolio did not receive as high ratings. Areas for improvement highlighted in free-text answers were: adding more face-to-face sessions and discussions about on-call scenarios.

**Conclusion:**

Transition into NHS can be a challenging experience for doctors at all stages of their careers. However, early intervention and a comprehensive induction programme appear to have had a positive impact on new trainee doctors’ confidence and preparedness for work. The programme's structure and the sessions content were modified in response to feedback. Additionally, individual support sessions and a writing group were organised for trainees and SAS doctors.

